# Supplementary material for: A Low-Cost Ecofriendly Oxidation Process to Manufacture High-Performance Polymeric Biosurfactants Derived from Municipal Biowaste
Source: Polymers (Basel). 2024 May 23;16(11):1479. doi: 10.3390/polym16111479 (PMC11174893; doi:10.3390/polym16111479)
Supplement: Supplementary file 1 [file polymers-16-01479-s001.zip › polymers-2939017-supplementary.pdf]

A Low-Cost Ecofriendly Oxidation Process to Manufacture High-Performance Polymeric Biosurfactants Derived from Municipal Biowaste

**Elio Padoan [1], Francesco Contillo [2], Matteo Marafante [3], Enzo Montoneri [1,*], Matteo Francavilla [2], Silvia Berto [3]
and Andrea Baglieri [4]**

[1] Dipartimento di Scienze Agrarie, Forestali e Alimentari, Università di Torino, 10095 Grugliasco, Italy; elio.padoan@unito.it
[2] STAR Integrated Research Unit, Università di Foggia, 71121 Foggia, Italy; francesco.contillo@unifg.it (F.C.); matteo.francavilla@unifg.it (M.F.)
[3] Dipartimento di Chimica, Università di Torino, 10125 Torino, Italy; matteo.marafante@unito.it (M.M.); silvia.berto@unito.it (S.B.)
[4] Dipartimento di Scienze delle Produzioni Agrarie e Alimentari, Università di Catania, Via S. Sofia 98,
95123 Catania, Italy; abaglie@unict.it
* Correspondence: enzo.montoneri@gmail.com

Table S1. Chemical composition data for products reported in Figures 3-5, 10 of main manuscript for products obtained by irradiation of SBP

	Not irradiated SBP	No.0-R200	No.0-R100	No.3-R200	No.3-R100	No.4-R200	No.4-R100	ozonised R750	ozonised R150
Af	37.6	49.2	61.6	53.4	39.8	48.4	49.9	78.8	76.9
NR+OMe	7.9	8.1	10.1	10	5.5	8.2	7.2	1.2	2.6
OR	15.6	10.4	7.5	10.1	7.3	12.4	8	3.3	1.5
OCO	4.6	2.3	0.44	2.1	3.7	2.8	2.5	3	2.6
Ph	14.4	12.5	4.6	7.6	5.4	10.2	5.2	4.9	4
PhOY	5.5	3.9	1.4	0.88	5.7	4.2	1.7	4.1	3.9
COX	14.4	13.6	14.3	15.9	32.6	13.9	25.4	4.8	8.4
Af/COX	2.61	3.61	4.31	3.36	1.22	3.48	1.96	16.4	9.15
Af+COX	52.0	62.8	75.9	69.3	72.4	62.3	75.3	52	62.8
Ph+PhOY	19.9	16.4	6.00	8.48	11.1	14.4	6.9	19.9	16.4
(Af+COX)/(Ph+PhOY)	2.61	3.83	12.6	8.17	6.52	4.33	20.9	9.29	10.8
Af+NR+OMe+OR+OCO+COX	80.1	93.6	93.9	91.5	88.9	85.7	93	91.1	92.0
Af +OR+OCO+COX	72.2	75.5	83.8	81.5	83.4	77.5	85.8	89.9	89.4
(Af+NR+OMe+OR+OCO+COX)/(Ph+PhOY)	4.02	5.10	15.7	10.8	8.01	5.95	13.5	10.1	11.6
(Af +OR+OCO+COX)/(Ph+PhOY)	3.63	4.60	14.0	9.61	7.51	5.38	12.4	9.99	11.3

Figure S1. Experimental apparatus for the ultrafiltration of the samples.

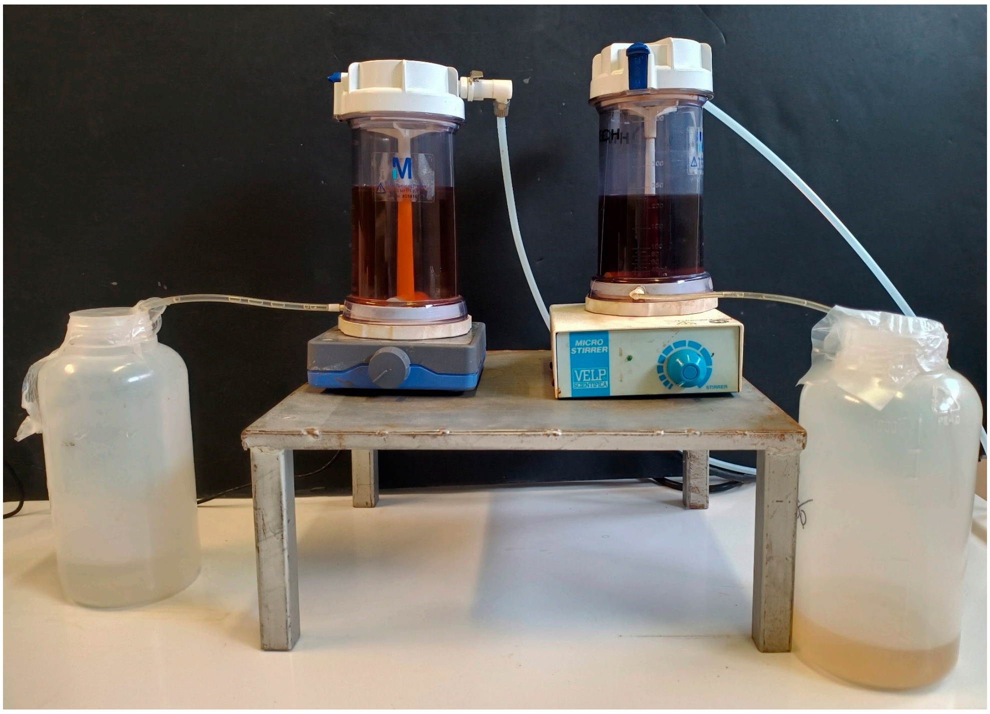

Calculation of HLB values (see Table S2) for products obtained by irradiation of SBP

HLB values were calculated according to the following equations 1-3:

$$Ci = Cbai\ Corgdm\ Cdm \quad (1)$$

$$HLBi = Ci\ gni \quad (2)$$

$$HLBT = \Sigma\ HLBi \quad (3)$$

In equations 1-3, Ci is the content of the individual C type in the 2 g/L solution used for the surface tension measurements; Cbai is the individual C type % band area measured in the recorded 13 C NMR spectrum (Figure 3); Corgdm is the product organic C concentration in the recovered mass of the product; Cdm is the 2 g/L dry matter content of the product in the solution used to obtain the surface tension values; gni are the individual group number values for each C type reported in the specialised literature (30, 33, 34); HLBi is the contribution of the individual C type to the total product HLB value; HLBT is the total HLB value calculated from the sum of the HLBi value (equation 3). References are as in the main text.

Table S2. Calculation of HLB values for products obtained by irradiation of SBP

		Not irradiated SBP			*No.0-R200*			*No.0-R100*			*NoO.3-R200*			*No.3-R100*		
C type	**gn**	**Cbai**	**Cì**	**HLBi**	**Cbai**	**Ci**	**HLBi**	**Cbai**	**Ci**	**HLBi**	**Cbai**	**Ci**	**HLBi**		**Ci**	**HLBi**
Af	0.475	37.6	0.269	0.128	49.2	0.441	0.209	61.6	0.428	0.203	53.4	0.470	0.223	39.8	0.213	0.101
NR+OMe	9.4	7.9	0.057	0.532	8.1	0.073	0.682	10.1	0.070	0.659	10	0.088	0.827	5.5	0.029	0.277
OR	1.9	15.6	0.112	0.212	10.4	0.093	0.177	7.5	0.052	0.099	10.1	0.070	0.133	7.3	0.039	0.074
OCO	1.8	4.6	0.033	0.059	2.3	0.021	0.037	0.44	0.003	0.005	2.1	0.018	0.033	3.7	0.020	0.036
Ph	0.475	14.4	0.103	0.049	12.5	0.112	0.053	4.6	0.032	0.015	7.6	0.067	0.032	5.4	0.029	0.014
PhOY	1.3	5.5	0.039	0.051	3.9	0.035	0.045	1.4	0.010	0.013	0.88	0.008	0.010	5.7	0.031	0.040
COOK+CON	2.2	14.4	0.103	0.227	13.6	0.122	0.268	14.3	0.099	0.218	15.9	0.140	0.308	32.6	0.175	0.384
HLBT				1.258			1.472			1.212			1.567			0.926
Corgdm		35.8			44.8			34.7			44			26.8		
Cdm		2			2			2			2			2		

	**No.4-R200**			**No.4-R100**		
C type	**Cbai**	**Ci**	**HLBi**	**Cbai**	**Ci**	**HLBi**
Af	48.4	0.452	0.215	49.9	0.325	0.155
NR+OMe	8.2	0.077	0.720	7.2	0.047	0.441
OR	12.4	0.116	0.220	8.0	0.052	0.099
OCO	2.8	0.026	0.047	2.5	0.016	0.029
Ph	10.2	0.095	0.045	5.2	0.034	0.016
PhOY	4.2	0.039	0.051	1.7	0.011	0.014
COOK+CON	13.9	0.130	0.286	25.4	0.166	0.364
HLBT			1.584			1.119
Corgdm	46.7			32.6		
Cdm	2			2		

Table S3. Samples and data for Figure 12

Sample	References as in main paper	(Af+OR+OCO+COOH)/COOH, mol/mol ratio	γ, mN/m
water	This work	0	70
SBP not irradiated	This work	6.56	49
No.0-R200	This work	12.0	34.3
No.0-R100	This work	9.37	40.9
No.3-R200	This work	7.31	44.3
N0.3-R100	This work	13.6	45
No.4-R200	This work	13.9	34.9
CVD[a]	20b	4.48	58.9
CVD R35[b]	20b	4.49	59.8
CVDoz R35[b]	20b	7.84	51.9
CVDoz R5[c]	20b	5.06	66.6
CVDoz P5[c]	20b	1.60	70.5
CV[d]	20b	11.2	58.9
CV R35[e]	20b	2.67	59.2
CVoz R35[f]	20b	6.74	48.5
CVoz R20[f]	20b	2.64	63.2
CVoz R5[f]	20b	2.35	70.5
MW CVD 60°C R5[g]	20c	6.37	63.5
MW CVD 100°C R5[g]	20c	6.44	58.5
MW CVD 150°C R5[g]	20c	5.73	52.1

[a] CVD = crude product obtained by hydrolysis at 60 °C from green-food waste compost. [b]Retentate at 35 kDa (R35) from CVD ultrafiltration. [c]Retentates at 35 kDa (R35) and 5 kDa (R5) and permeate at 5 kDa (P5) from ozonised CVD (CVDoz) ultrafiltration. [d]CV = crude product obtained by hydrolysis at 60 °C from green waste compost. [e]Retentate at 35 kDa (R35) from CV ultrafiltration. [f]Retentates at 35 kDa (R35), 20 kDa (R20) and 5 kDa (R5) from ozonised CV (CVoz) ultrafiltration. [g]Retentate at 5 kDa (R5) from crude CVD obtained by hydrolysis at 60, 100 and 150 °C carried out in microwave (MW) reactor.

Table S4. Data of individual C types and functional groups for products in Figure 12 of main manuscript.

	**Af**	**NR+OME**	**OR**	**OCO**	**Ph**	**PhOH**	**COOH**	**CON**	**PhOR(Ar)**
Water	0.0	0.0	0.0	0.0	0.0	0.0	0.0	0.0	0.0
SBP	37.6	7.9	15.6	4.6	14.4	2.3	10.4	4	3.1
No.0-R200	49.2	8.1	10.4	2.3	12.5	1.3	5.6	8	2.6
No.0-R100	61.6	10.1	7.5	0.4	4.6	1.2	8.3	6.05	0.2
No.3-R200	53.4	10	10.1	2.1	7.6	0.7	5.2	10.7	0.2
No.3-R100	39.8	5.5	7.3	3.7	5.4	2.2	10.5	22.1	3.5
No.4-R200	48.4	8.2	12.4	2.8	10.2	1	4.9	9	3.2
CVD	40.9	7.3	14.2	3.9	18.2	6.6	17.0	0.0	11.6
Rcvd35	22.5	9.3	8.8	3.4	33.1	1.8	9.9	13.0	31.3
Rcvdoz35	62.9	5.5	8.6	4.9	5.2	5.7	11.2	1.7	0.0
Rcvdoz5	38.9	11.5	2.6	5.8	5.6	3.1	11.7	24.0	2.5
Pcvdoz5	16.6	5.5	15.8	2.4	2.4	11.8	58.2	0.0	0.0
CV	36.9	7.2	13.2	4.2	20.2	5.1	14.5	3.8	15.1
Rcv35	26.7	8.3	8.1	2.6	31.7	11.7	22.4	0.2	20.0
Rcvoz35	49.9	10.1	13.5	3.1	5.3	1.9	11.6	6.5	3.4
Rcvoz20	21.5	11.4	17.7	3.1	4.1	10.4	25.8	16.4	0.0
Rcvoz5	17.9	9.6	19.5	2.9	1.9	12.6	29.7	18.5	0.0
MW CVD 60	37.0	8.3	16.3	4.8	12.9	1.6	10.8	4.7	3.6
MW CVD 100	43.3	7.9	9.1	3.1	14.2	1.5	10.2	7.1	3.7
MW CVD 150	44.1	8.6	8.5	2.7	16.9	1.8	11.7	1.5	4.1

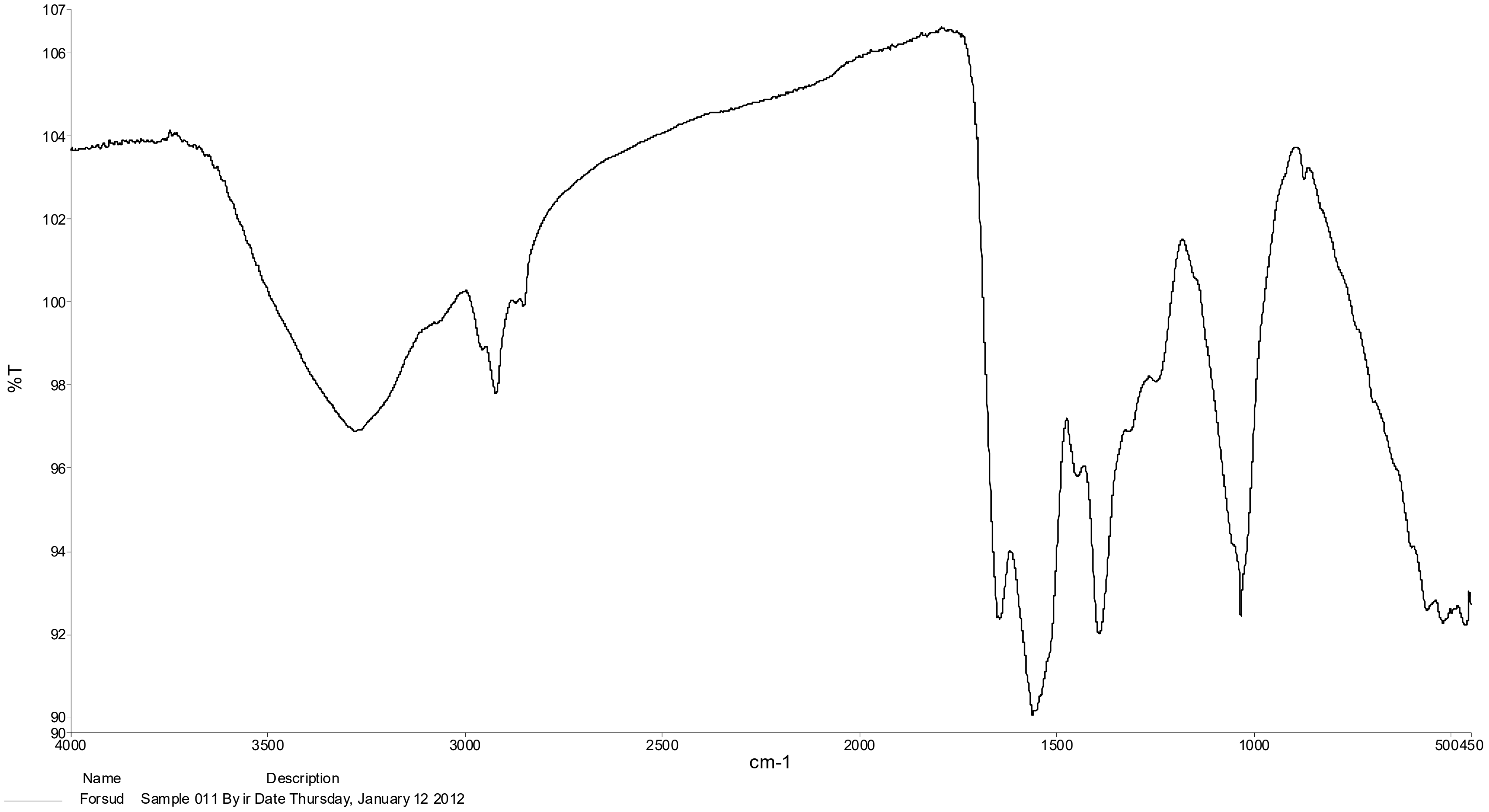
%T
107
106
104
102
100
98
96
94
92
90
90
4000
3500
3000
2500
2000
1500
1000
500450
cm-1
Name
Description
Forsud
Sample 011 By ir Date Thursday, January 12 2012

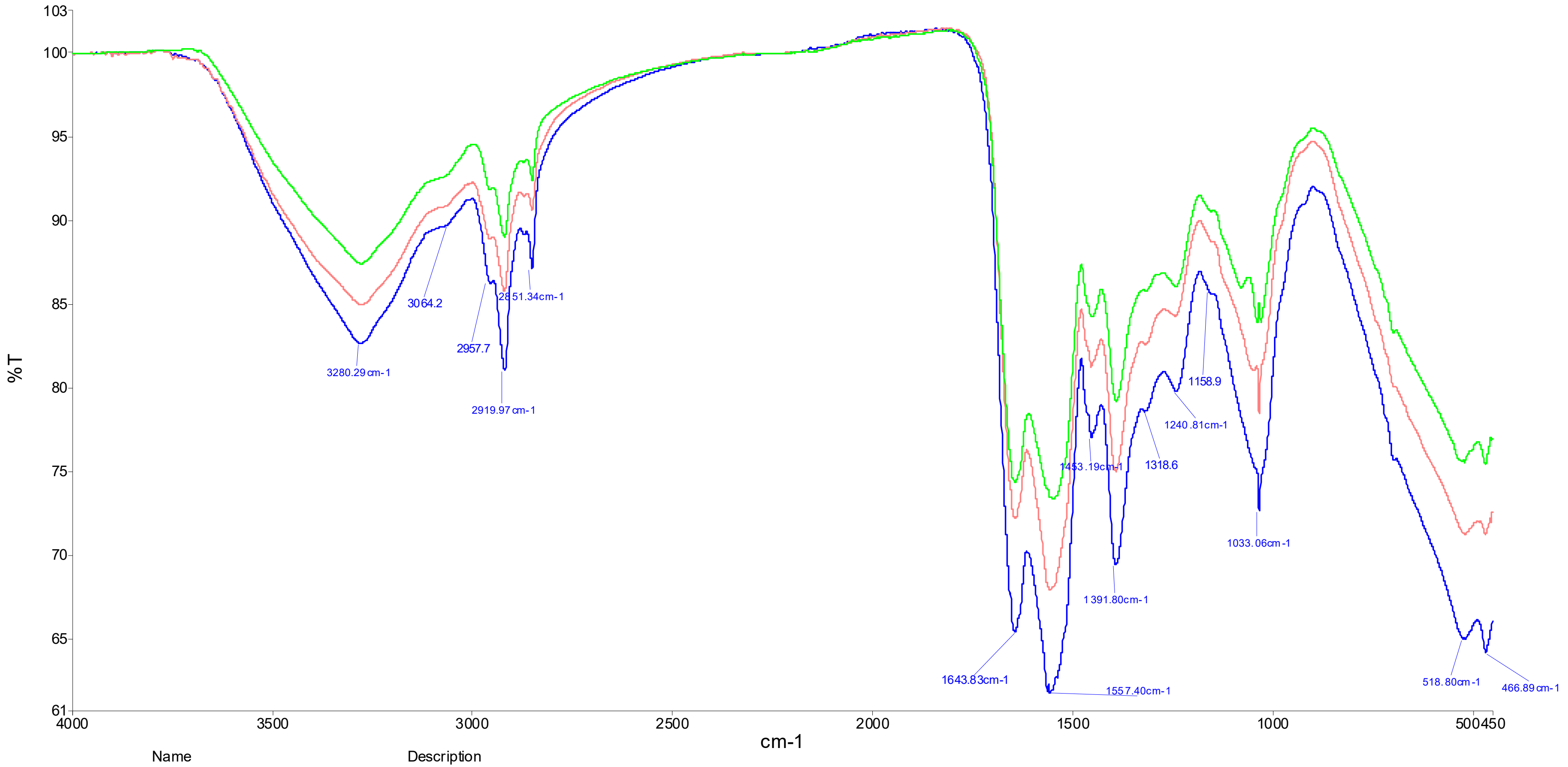

Name	Description
FotOx4_CutOff 200KDa_1	Sample 012 By ir Date Friday, January 13 2012
Controllo_CutOff200KDa_1	Sample 007 By ir Date Friday, January 13 2012
FotOx3 ut Off 200 kDa_1	Sample 003 By ir Date Monday, February 14 2022

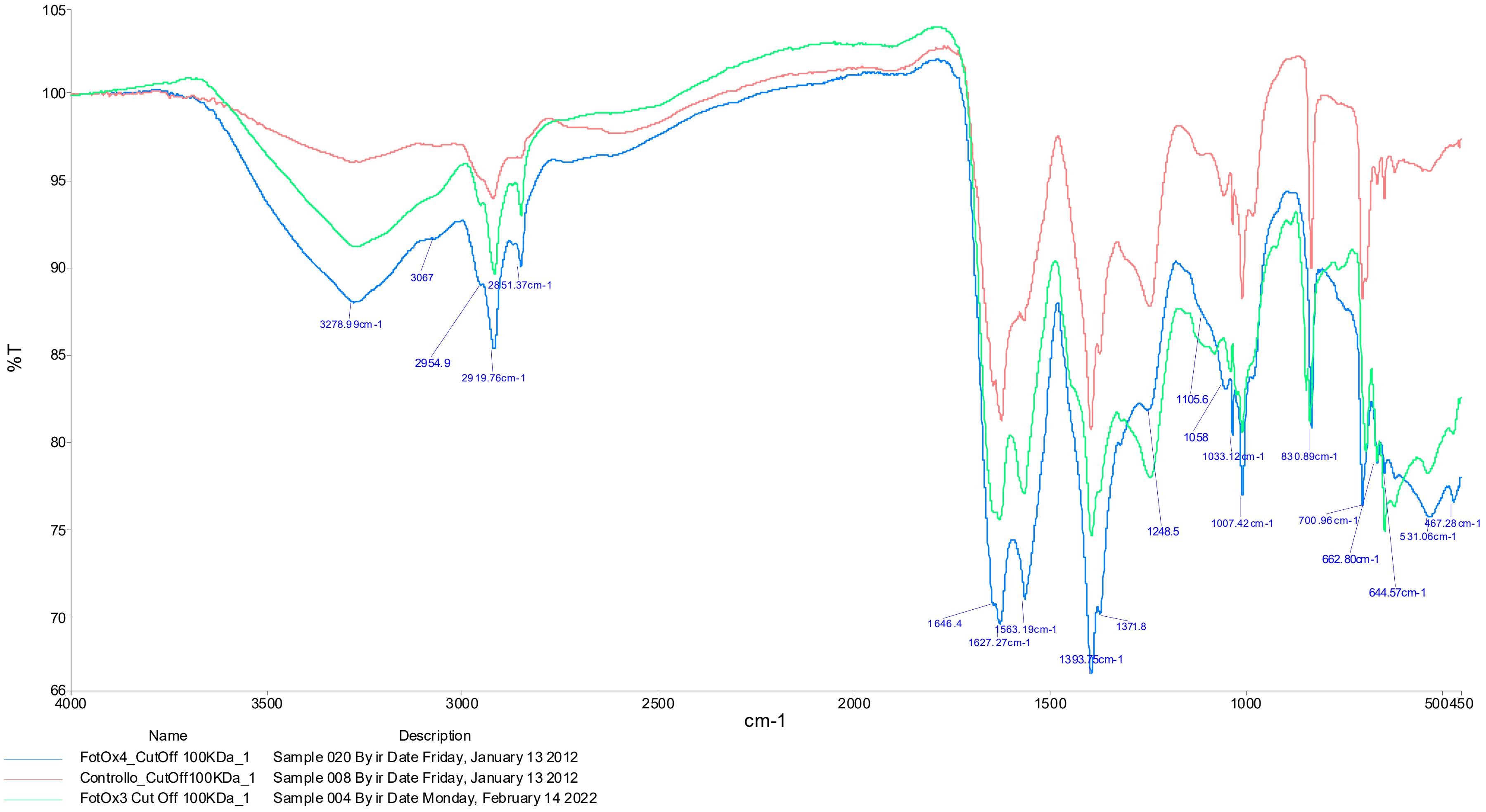
105
100
95
90
85
80
75
70
66
%T
4000
3500
3000
2500
2000
1500
1000
500450
cm-1
3278.99cm-1
3067
2954.9
2919.76cm-1
2851.37cm-1
1646.4
1627.27cm-1
1563.19cm-1
1393.75cm-1
1371.8
1248.5
1105.6
1058
1033.12cm-1
1007.42cm-1
830.89cm-1
700.96cm-1
662.80cm-1
644.57cm-1
531.06cm-1
467.28cm-1
Name
Description
FotOx4_CutOff 100KDa_1
Sample 020 By ir Date Friday, January 13 2012
Controllo_CutOff100KDa_1
Sample 008 By ir Date Friday, January 13 2012
FotOx3 Cut Off 100KDa_1
Sample 004 By ir Date Monday, February 14 2022

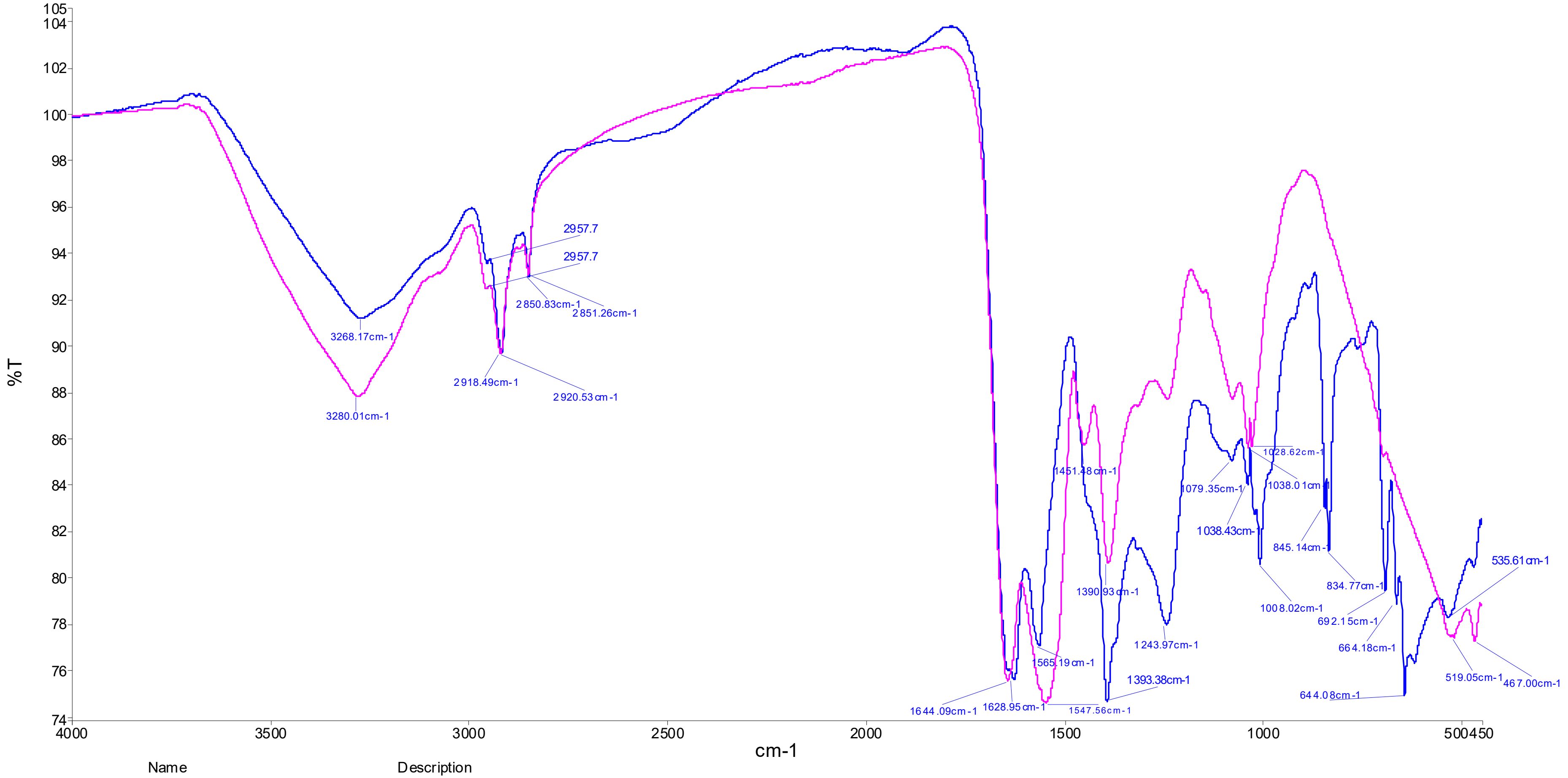

Name	Description
FotOx3 Cut Off 100KDa_1	Sample 004 By ir Date Monday, February 14 2022
FotOx3 ut Off 200 kDa_1	Sample 003 By ir Date Monday, February 14 2022

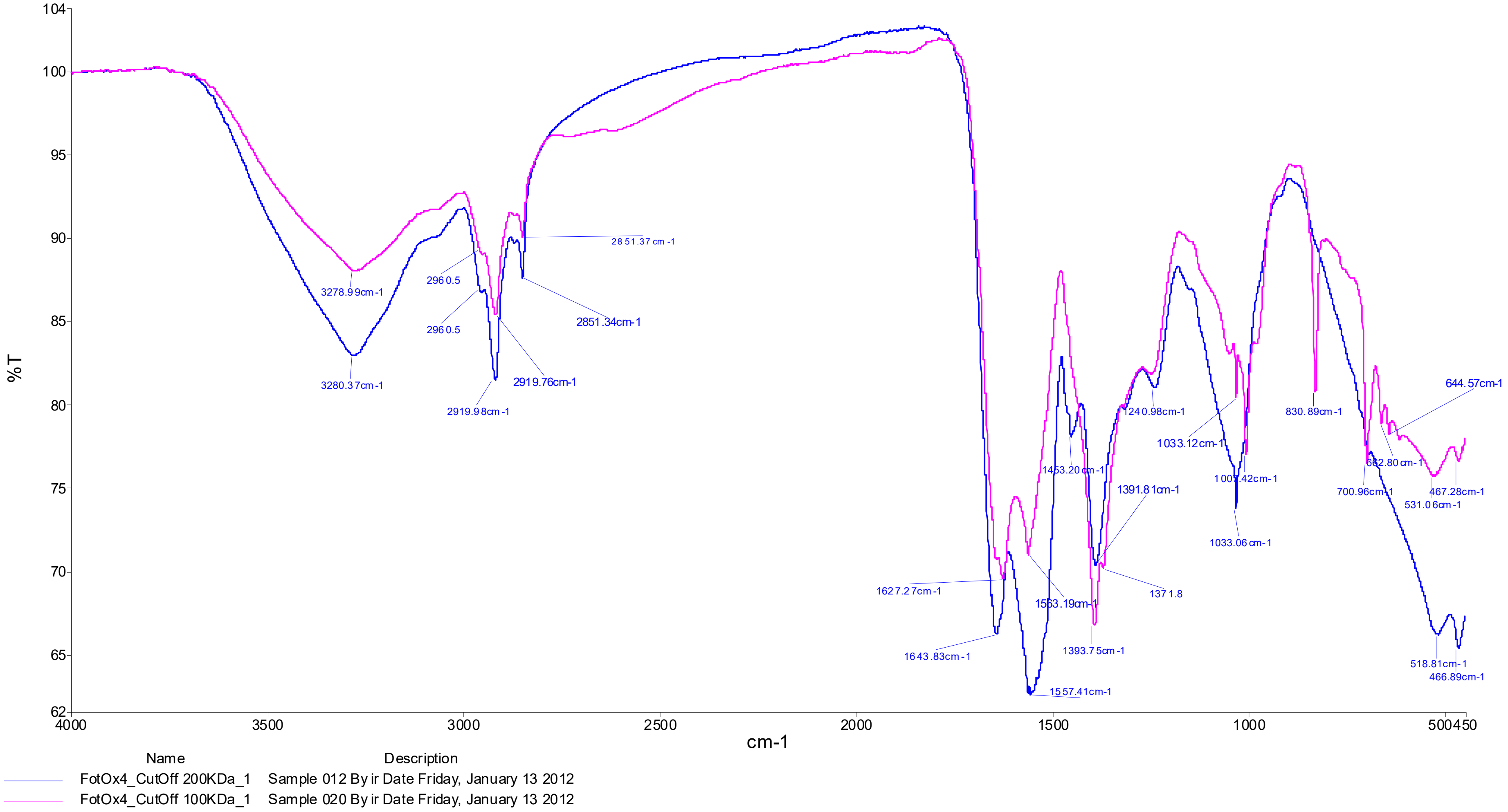

Figure S2. FTIR Spectra for produdcts obtained from SBP (Table 7 in the main manuscript), from top to bottom according to the products identification in Table 7: not irradiated SBP (Forsud), No.0-R200 (controllo_CutOff200kDa), No.3-R200 (FotOx3cutoff200kDa), No.4-R200 (FotOx4cutoff200kDa), No.0-R100 (controllo_CutOff100kDa), No.3-R100 (FotOx3cutoff100kDa), No.4-R100 (FotOx4cutoff100kDa).
